# Tetracyclines resistance in *Mycoplasma* and *Ureaplasma* urogenital isolates derived from human: a systematic review and meta-analysis

**DOI:** 10.1186/s12941-023-00628-5

**Published:** 2023-09-11

**Authors:** Xiaoyan Wen, Mojgan Sarabi Nobakht, Yue Yang, Ebrahim Kouhsari, Sara Hajilari, Matin Zafar Shakourzadeh, Khalil Azizian

**Affiliations:** 1 Urology Department of Affiliated Hospital of Chengdu University, Chengdu, 610000 Sichuan China; 2 Department of Microbiology, Faculty of Basic Sciences, Islamic Azad University, Sirjan Branch, Sirjan, Iran; 3 Laboratory Sciences Research Center, Golestan University of Medical Sciences, Gorgan, Iran; 4 Department of Microbiology, Faculty of Medicine, Kurdistan University of Medical Sciences, Sanandaj, Iran

**Keywords:** *Mycoplasma*, *Ureaplasma*, Antimicrobial resistance, Tetracyclines resistance, Systematic review, Meta-analysis

## Abstract

**Background:**

Urogenital *Mycoplasma* infections are considered an important public health problem, owing to the presence of antibiotic resistance or decreased susceptibility, the treatment options are limited.

**Objective:**

Therefore, this meta-analysis aimed to estimate resistance rates of genital *Mycoplasmas* to tetracyclines (tetracycline, doxycycline, and minocycline).

**Methods:**

We searched the relevant published studies in PubMed, Scopus, and Embase until 3, March 2022. All statistical analyses were carried out using the statistical package R.

**Results:**

The 26 studies included in the analysis were performed in 15 countries. In the metadata, the proportions of tetracycline, doxycycline, and minocycline resistance in *Mycoplasma* and *Ureaplasma* urogenital isolates were reported 14.2% (95% CI 8.2–23.2%), 5% (95% CI 3–8.1%), and 11.9% (95% CI 6.3–21.5%), respectively. According to the meta-regression, the tetracycline and minocycline resistance rate decreased over time. Although, the doxycycline resistance rate increased over time. There was a statistically significant difference in the tetracyclines resistance rates between different continents/countries (*P* < 0.05).

**Conclusion:**

The prevalence rate and antibiotic susceptibility profiles vary geographically. Therefore, rigorous or improved antimicrobial stewardship, contact tracing, and enhanced intensive surveillance systems are necessitated for preventing the emergence and further spreading of tetracyclines resistance in genital *Mycoplasmas.*

**Supplementary Information:**

The online version contains supplementary material available at 10.1186/s12941-023-00628-5.

## Introduction

Mollicutes class contains the *Mycoplasma* genus is the smallest free-living and self-replicating micro-organisms [1, 2]. The *Mycoplasma* genus is divided into respiratory and genital Mycoplasmas. The genital Mycoplasmas include *Mycoplasma hominis* and *Ureaplasma parvum* and *Ureaplasma urealyticum*, which are frequently found in the lower urogenital tract of sexually active healthy adults [3]. The prevalence rate of Mycoplasmas in the genital tract of sexually active Men and women is approximately 5–20% and 40–80%, respectively [4]. These microorganisms identified as causative agents of several urogenital disorders in adults and children and have global distribution [3, 5]. These disorders include bacterial vaginosis, non-gonococcal urethritis (NGU), cystitis, pyelonephritis, endometritis, and cervicitis [3, 6, 7]. Also, the caused infection by these organisms may be symptomatic or asymptomatic. It is proven, that asymptomatic infections are related to increased risks of pelvic inflammatory disease (PID), infertility adverse pregnancy outcomes [8]. In another hand, because of serious complications in newborns and immunocompromised patients, adequate treatment and bacterial eradication is crucial.

Owing to the absence of the cell wall in Mycoplasmas, these bacteria have a natural resistance to antibiotics affecting on the cell wall, like glycopeptides (vancomycin and teicoplanin) and beta-lactams (penicillins and cephalosporins) [9, 10]. However, these bacteria are susceptible to antibiotics that interfere with bacterial DNA replication (quinolones) and bacterial protein synthesis (tetracyclines and macrolides). Therefore, currently, the abovementioned antibiotics are known as the first-line treatment and primary choice in the empirical treatment caused by Mycoplasmas [5]. But *M. hominis* has developed resistance to quinolones globally [11]. Also, resistance owing to mutations in 23S rRNA caused resistance to erythromycin and azithromycin [12, 13]. Thus, in Mycoplasmas infections tetracyclines class (tetracycline, doxycycline, and minocycline) is recommended as a primary choice of chemical therapy, except for pregnant women and neonates [14].

The extensive overuse of tetracyclines has led to a gradual rise in resistance of Mycoplasmas and, tetracyclines resistance strain is a growing phenomenon worldwide [1, 7]. While, the rising rates of resistant isolates have been noted, a limited number of studies performed on the status of resistance to tetracyclines. Therefore, this systematic meta-analysis was conducted to survey the status of resistance to the tetracycline class, (tetracycline, doxycycline and minocycline) by analyzing the related published studies.

## Methods

This review is reported in accordance with the Preferred Reporting Items for Systematic Reviews and Meta-Analyses guidelines (PRISMA) [15].

### Search strategy and study selection

We systematically searched for relevant articles in PubMed, Scopus, and Embase (Until August 2021) by using the related keywords: ("*Mycoplasma*" OR "*Mycoplasma hominis*" OR "*M. hominis*" OR "*Mycoplasma genitalium*" OR "*M. genitalium*" OR "*Ureaplasma*" OR "*Ureaplasma urealyticum*" OR "*U. urealyticum*" OR "*Ureaplasma parvum*" OR "*U. parvum*" AND "antibiotic" OR "antimicrobial" OR "tetracycline" OR "doxycycline" OR "minocycline" AND "resistance" OR "resistant") in the Title/Abstract/Keywords fields. No limitation was used while searching databases. The search strategy was designed and conducted by study investigators. References lists of all related studies were also reviewed for any other related publication. The records found through database searching were merged, and the duplicates were removed using EndNote X9 (Thomson Reuters, New York, NY, USA). One of the team researchers randomly evaluated the search results and confirmed that no relevant study had been ignored. The authors did all these steps, and any disagreements about article selection were resolved through discussion. References from reviewed articles were also searched for more information.

### Inclusion and exclusion criteria

The included studies met the following criteria: (1) original study that investigated tetracyclines (tetracycline, doxycycline, and minocycline) resistance in *Mycoplasma* and *Ureaplasma* urogenital isolates derived from human; (2) peer-reviewed articles published in English between 1978 and 2021; (3) specified the total number of tested *Mycoplasma* and *Ureaplasma* isolates; (4) reported the tetracyclines resistance rate in *Mycoplasma* and *Ureaplasma* isolates. The exclusion criteria were as follows: (1) studies that contained duplicate data or were overlapping articles; (2) no clinical *Mycoplasma* and *Ureaplasma* urogenital isolates; (3) reported antibiotic resistance of mycoplasmas other than *M. hominis*, *U. urealyticum/parvum*; (4) studied resistance to antibiotics other than tetracyclines; (5) reviews, meta-analysis and/or systematic review, and conference abstracts; (5) resistance rates were not clearly presented or reported.

### Data extraction

The following items were extracted from each included study: first author, publication year, continent, country, participants’ characteristics (gender (male/female), type of complication) number of clinical *M. hominis, U. urealyticum,* and* U. parvum* isolates*,* number of tetracyclines resistance rate in *M. hominis, U. urealyticum, and U. parvum* isolates, and antimicrobial susceptibility testing (AST; MIC-Based methods and *Mycoplasma* kits). Data were collected by two independent examiners and verified by another researcher. The resistance rate was expressed as the number of resistant isolates divided by the total number of isolates tested.

### Quality assessment

The quality of the included studies was assessed by two reviewers separately using an adapted version of the tool proposed by the Newcastle–Ottawa assessment scale adapted for cross-sectional studies [16]. A score ranging from 0 to 8 points was attributed to each study (≥ 6 points: high quality, ≤ 5 points: low quality). A higher score indicated a higher study quality. A third reviewer adjudicated in any cases where there was disagreement.

### Statistical analysis

The studies presenting raw data on tetracycline, doxycycline, and minocycline resistance in *Mycoplasma* and *Ureaplasma* urogenital isolates derived from human were included in the meta-analysis that was carried out using the meta-prop [17] command in R statistical software on all prevalence statistics by antibiotic, region (continents/countries) and AST. The meta-analysis results consist of a prevalence statistic with 95% confidence intervals calculated from the weighted prevalence statistics for all the studies in the specified sub-group by antibiotic, region (continents/countries), and AST. Meta-regression models were used to check for changing antibiotic resistance over time and male/female ratio. Publication bias was assessed using Egger's and begg's test. All statistical interpretations were reported on a 95% confidence interval (CI) basis. All statistical analyses were carried out using the statistical package R 3.6.0 (R Foundation for Statistical Computing: Vienna, Austria) [18].

### Study outcomes

The main outcome of interest was the proportion of tetracycline, doxycycline, and minocycline resistance in *Mycoplasma* and *Ureaplasma* urogenital isolates derived from human. A subgroup analysis was performed; [1] subgroup analyses were then employed by areas (continents/countries), [3] and AST. Meta-regression models were used to check for changing antibiotic resistance over time and male/female ratio.

## Results

### Systematic literature search

**Fig. 1:**
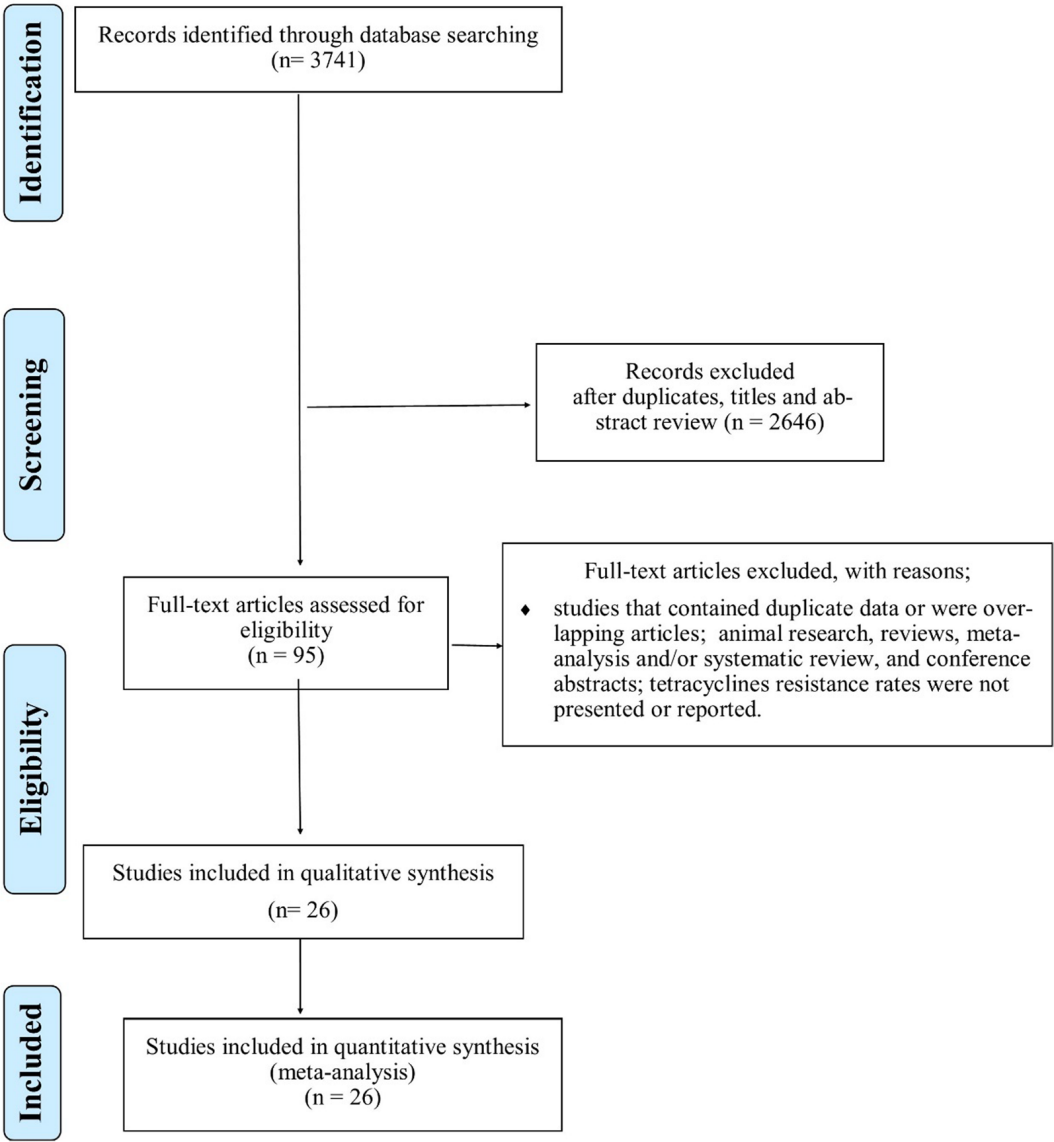
Flow chart of study selection

A total of 2741 records were identified in the initial search. From these, 2646 articles were excluded after an initial screening of the title and abstract due to their irrelevance and duplication. The full texts of the remaining 95 articles were reviewed (Fig. 1). From the 95 articles, 57 were excluded for the following reasons: (1) studies that contained duplicate data or were overlapping articles; (2) animal research, reviews, meta-analysis and/or systematic review, and conference abstracts; (3) tetracyclines resistance rates were not presented or reported. Finally, the 26 studies included [19–44] were published between 1978 and 2021 (Additional file 1: Table S1).

### Characteristics of included studies

**Fig. 2:**
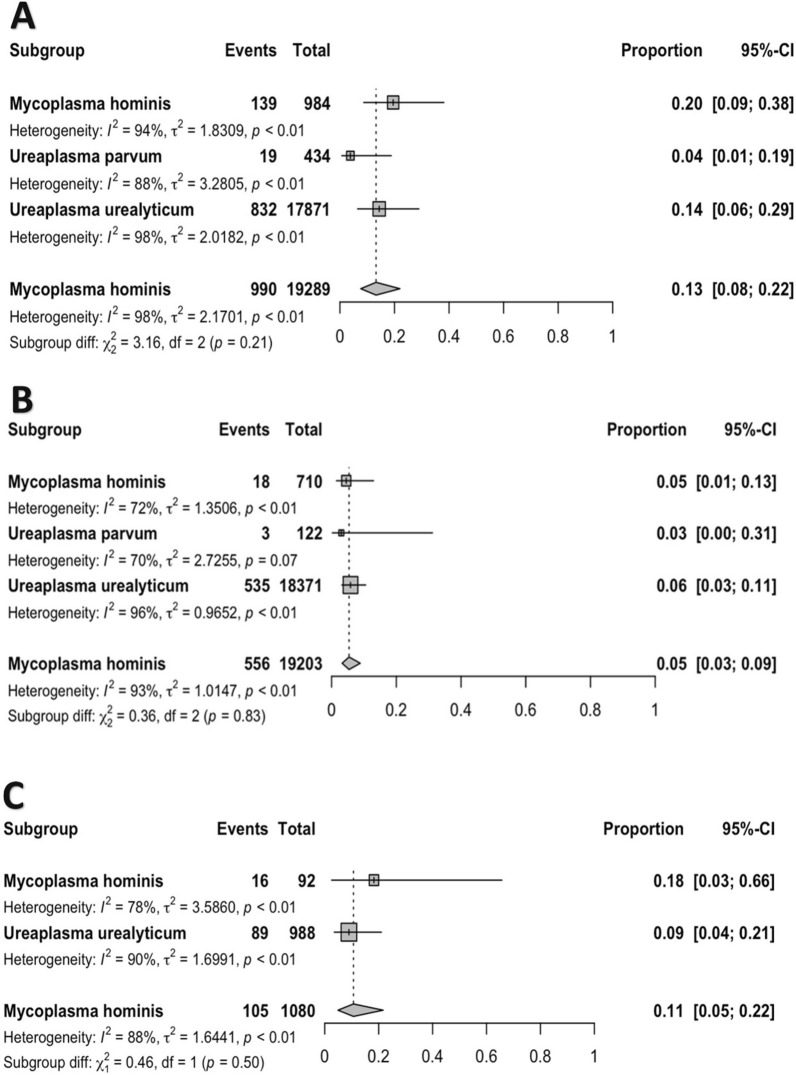
Forest plot proportions of tetracycline (**A**), doxycycline (**B**) and minocycline (**C**) resistance isolates to selected included antibiotics

The 26 studies included in the analysis were performed in 15 countries. The majority of the studies included in the meta-analysis revealed the resistance to tetracycline (20 reports) followed by doxycycline (15 reports), and minocycline (10 reports). Figure 2 showing forest plot proportions of resistance isolates to selected included antibiotics. The proportion of each antibiotic and the subgroup analyses by continent/countries, genus, species, and AST, are shown in Table 1. The trends in resistance rates to tetracycline, doxycycline, and minocycline are summarised below.

**Table 1:** Prevalence of tetracyclines resistance in *Mycoplasma* and *Ureaplasma* urogenital isolates

Antibiotic	Subgroups	Variables	n, N	Proportion (LCI, HCI)	I^2^ (%)	*P*. value	Egger test
Tetracycline	Overall		1024, 19,424	0.142 (0.082, 0.232)	97.43	0.048	0.0310
	Continent	North America	46, 146	0.095 (0.004, 0.712)	91.50	< 0.001	
		Asia	695, 17,001	0.160 (0.075, 0.306)	96.55	< 0.001	
		Europe	104, 1715	0.059 (0.022, 0.149)	90.86	< 0.001	
		Africa	102, 358	0.281 (0.177, 0.416)	81.26	< 0.001	
		South America	77, 204	0.436 (0.206, 0.697)	90.96	< 0.001	
	Country	USA	46, 146	0.095 (0.004, 0.712)	91.50	< 0.001	
		China	652, 16,907	0.045 (0.021, 0.096)	96.60	< 0.001	
		Italy	60, 934	0.484 (0.001, 0.999)	97.84	< 0.001	
		Greece	0, 187	0.005 (0.001, 0.037)	0.00	< 0.001	
		Iraq	43, 94	0.457 (0.140, 0.813)	85.02	< 0.001	
		Senegal	75, 243	0.342 (0.190, 0.536)	86.06	< 0.001	
		Tunisia	22, 65	0.338 (0.234, 0.461)	0.00	< 0.001	
		Turkey	0, 30	0.016 (0.001, 0.211)	0.00	< 0.001	
		England	12, 81	0.148 (0.086, 0.243)	0.00	< 0.001	
		Cuba	77, 204	0.436 (0.206, 0.697)	90.96	< 0.001	
		Romania	4, 50	0.080 (0.030, 0.195)	0.00	< 0.001	
		Spain	1, 250	0.006 (0.001, 0.031)	0.00	< 0.001	
		South Africa	5, 50	0.100 (0.042, 0.219)	0.00	< 0.001	
		France	27, 183	0.148 (0.103, 0.207)	0.00	< 0.001	
	Order	Mycoplasmatales	1024, 19,424	0.142 (0.082, 0.232)	97.43	< 0.001	
	Genera	*Ureaplasma*	885, 18,440	0.119 (0.060, 0.220)	97.59	< 0.001	
		*Mycoplasma*	139, 984	0.195 (0.087, 0.381)	93.78	< 0.001	
	Species	*Ureaplasma urealyticum*	832, 17,871	0.144 (0.065, 0.289)	98.32	< 0.001	
		*Mycoplasma hominis*	139, 984	0.195 (0.087, 0.381)	93.78	< 0.001	
		*Ureaplasma parvum*	19, 434	0.038 (0.007, 0.188)	88.26	< 0.001	
	AST	MIC Based	132, 692	0.140 (0.055, 0.313)	91.06	< 0.001	
		*Mycoplasma* kit	892, 18,732	0.142 (0.075, 0.253)	97.90	< 0.001	
Doxycycline	Overall		556, 19,338	0.050 (0.030, 0.081)	92.73	< 0.001	0.0070
	Continent	Asia	470, 17,657	0.036 (0.021, 0.062)	89.23	< 0.001	
		Europe	15, 1151	0.015 (0.010, 0.025)	0.00	< 0.001	
	Country	Africa	36, 326	0.144 (0.052, 0.340)	81.14	< 0.001	
		South America	35, 204	0.172 (0.126, 0.230)	0.00	< 0.001	
		China	467, 17,622	0.033 (0.018, 0.058)	89.99	< 0.001	
		Italy	15, 934	0.017 (0.010, 0.027)	0.00	< 0.001	
		Greece	0, 187	0.005 (0.001, 0.037)	0.00	< 0.001	
		Cameron	8, 18	0.362 (0.053, 0.852)	59.29	< 0.001	
		Iraq	3, 35	0.086 (0.028, 0.234)	0.00	< 0.001	
		Senegal	28, 243	0.115 (0.081, 0.162)	0.00	< 0.001	
		Tunisia	0, 65	0.008 (0.000, 0.110)	0.00	< 0.001	
		Turkey	0, 30	0.016 (0.001, 0.211)	0.00	< 0.001	
		Cuba	35, 204	0.172 (0.126, 0.230)	0.00	< 0.001	
	Order	Mycoplasmatales	556, 19,338	0.050 (0.030, 0.081)	92.73	< 0.001	
	Genera	Ureaplasma	538, 18,628	0.050 (0.029, 0.086)	94.19	< 0.001	
		Mycoplasma	18, 710	0.045 (0.015, 0.130)	71.53	< 0.001	
	Species	*Ureaplasma urealyticum*	535, 18,371	0.058 (0.032, 0.105)	95.73	< 0.001	
		*Mycoplasma hominis*	18, 710	0.045 (0.015, 0.130)	71.53	< 0.001	
		*Ureaplasma parvum*	3, 122	0.030 (0.002, 0.312)	69.64	< 0.001	
	AST	Mycoplasma kit	549, 18,921	0.061 (0.035, 0.106)	94.68	< 0.001	
		MIC Based	7, 417	0.025 (0.011, 0.054)	22.04	< 0.001	
Minocycline	Overall		131, 1256	0.119 (0.063, 0.215)	87.34	0.789	0.6886
	Continent	Asia	57, 774	0.099 (0.029, 0.289)	86.18	< 0.001	
		Europe	27, 226	0.118 (0.031, 0.362)	87.96	< 0.001	
		South America	30, 204	0.147 (0.099, 0.212)	12.76	< 0.001	
		North America	17, 52	0.327 (0.214, 0.464)	0.00	< 0.001	
	Country	China	25, 715	0.039 (0.020, 0.073)	43.01	< 0.001	
		Iraq	32, 59	0.487 (0.025, 0.972)	87.18	< 0.001	
		Italy	12, 35	0.343 (0.206, 0.512)	0.00	< 0.001	
		Cuba	30, 204	0.147 (0.099, 0.212)	12.76	< 0.001	
		Romania	1, 50	0.020 (0.003, 0.129)	0.00	< 0.001	
		England	14, 141	0.099 (0.060, 0.161)	0.00	< 0.001	
		USA	17, 52	0.327 (0.214, 0.464)	0.00	< 0.001	
	Order	Mycoplasmatales	131, 1256	0.119 (0.063, 0.215)	87.34	< 0.001	
	Genera	*Ureaplasma*	115, 1164	0.109 (0.053, 0.213)	89.65	< 0.001	
		*Mycoplasma*	16, 92	0.182 (0.025, 0.657)	78.39	< 0.001	
	Species	*Ureaplasma urealyticum*	89, 988	0.090 (0.035, 0.210)	90.32	< 0.001	
		*Mycoplasma hominis*	16, 92	0.182 (0.025, 0.657)	78.39	< 0.001	
	AST	MIC-Based	44, 480	0.096 (0.030, 0.264)	89.45	< 0.001	
		*Mycoplasma* kit	87, 776	0.133 (0.059, 0.274)	85.45	< 0.001	

*K* Number of reports, *n* Number of resistant isolates, *N* Number of total isolates, *LCI* 95% Lower Confidence Interval, *HCI* 95% Higher Confidence Interval

*P*-value of difference between groups

### Tetracycline resistance

**Fig. 3:**
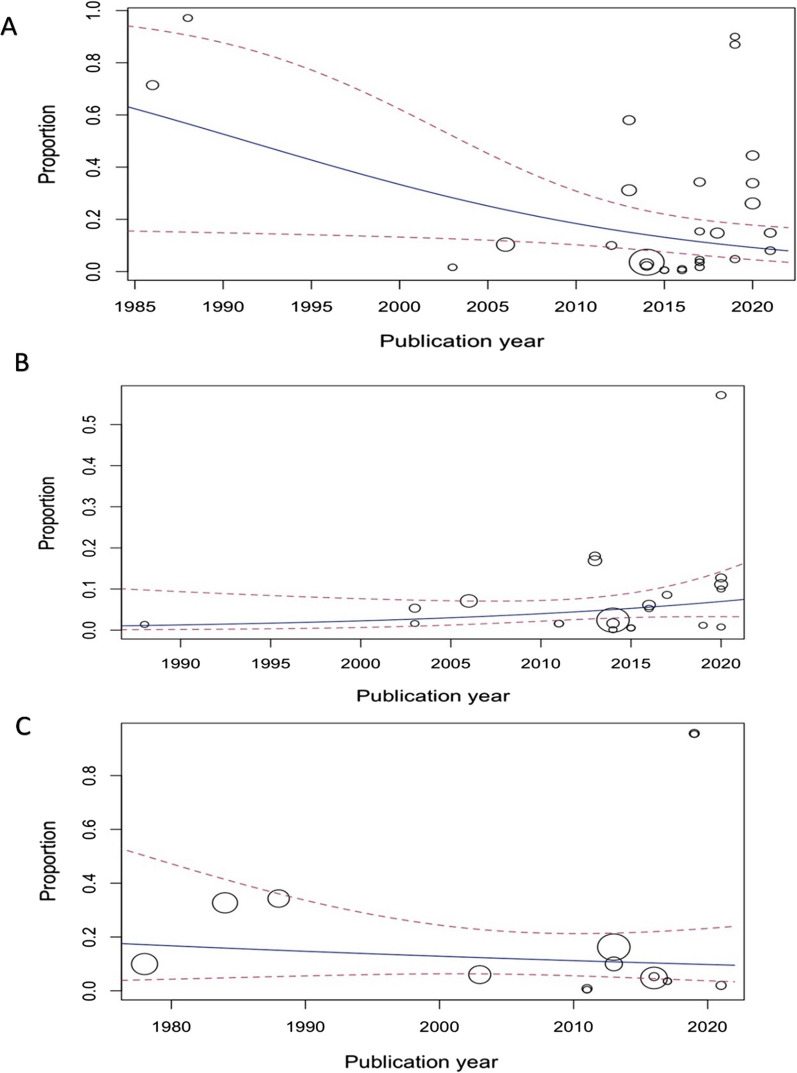
Meta-regression analysis for changes in the proportion of tetracycline (**A**), doxycycline (**B**) and minocycline (**C**) resistance to *Mycoplasma/Ureaplasma* urogenital isolates over time

The susceptibility to tetracycline was determined in 20 studies, including 19,424 *Mycoplasma* and *Ureaplasma* isolates [*M. hominis* (984 isolates), *U. urealyticum* (17,871 isolates), and *U. parvum* (434 isolates)]; the proportions were 20% (95% CI 9–38%), 14% (95% CI 6–29%), and 4% (95% CI 1–19%) in *M. hominis*, *U. urealyticum*, and *U. parvum*, respectively, with substantial heterogeneity (I^2^ > 88%; *P* = 0.21) was observed between included studies (Table 1, Fig. 2). Also, significant publication bias was detected (Egger rank correlation test, *P* = 0.0310). To analyze the trends for changes in the rate of tetracycline resistance in recent years, we performed a meta-regression analysis for changes in the proportion of tetracycline resistance to *Mycoplasma/Ureaplasma* urogenital isolates over time (Fig. 3). According to the meta-regression, the tetracycline resistance rate decreased over time (r = −0.080; 95% CI −0.153 to −0.007, *P* = 0.032). We performed a meta-regression analysis for changes in the proportion of tetracycline resistance in male/female ratio to *Mycoplasma*/*Ureaplasma* urogenital isolates. According to the meta-regression, the tetracycline resistance rate increased in male/female ratio (r = 0.012; 95% CI −0.011 to 0.036, *P* = 0.312). Among 14 countries (from 5 continents) reporting resistance data for tetracycline, 5 (35.7%) countries (Cuba, Iraq, Italy, Senegal, and Tunisia) reported that > 15% of isolates had tetracycline resistance. There was a statistically significant difference in the tetracycline resistance rates between different countries (*P* < 0.01) (Table 1). There was a statistically significant difference in the tetracycline resistance rates between different continents (*P* < 0.01), and this rate was higher in South America than Africa (44% vs 28%), Asia (44% vs 16%), North America (44% vs 10%), and Europe (44% vs 6%). No significant difference was found in the AST method (*P* = 0.98) (Table 1).

### Doxycycline resistance

The susceptibility to doxycycline was determined in 15 studies, including 19,203 *Mycoplasma* and *Ureaplasma* isolates [*M. hominis* (710 isolates), *U. urealyticum* (18,371 isolates), and *U. parvum* (122 isolates)]; the proportions were 5% (95% CI 1–13%), 6% (95% CI 3–11%), and 3% (95% CI 0–31%) in *M. hominis*, *U. urealyticum*, and *U. parvum*, respectively, with substantial heterogeneity (I^2^ = 93%; *P* = 0.83) was observed between included studies (Table 1, Fig. 2). Also, significant publication bias was detected (Egger rank correlation test, *P* = 0.0070). To analyze the trends for changes in the rate of doxycycline resistance in recent years, we performed a meta-regression analysis for changes in the proportion of tetracycline resistance to *Mycoplasma/Ureaplasma* urogenital isolates over time (Fig. 3). According to the meta-regression, the doxycycline resistance rate increased over time (*r* = 0.059; 95% CI −0.027 to 0.145, *P* = 0.180). We performed a meta-regression analysis for changes in the proportion of doxycycline resistance in male/female ratio to *Mycoplasma*/*Ureaplasma* urogenital isolates. According to the meta-regression, the doxycycline resistance rate decreased in male/female ratio (r = −0.021; 95% CI −0.052 to 0.011, *P* = 0.205). Among 10 countries (from 4 continents) reporting resistance data for tetracycline, 4 (25%) countries (Cuba, Iraq, Cameroon, and Senegal) reported that > 6% of isolates had doxycycline resistance. There was a statistically significant difference in the doxycycline resistance rates between different countries (*P* < 0.01) (Table 1). There was a statistically significant difference in the doxycycline resistance rates between different continents (*P* < 0.01), and this rate was higher in South America than Africa (17% vs 14%), Asia (17% vs 4%), and Europe (17% vs 2%). No significant difference was found in the AST method (*P* = 0.07) (Table 1).

### Minocycline resistance

The susceptibility to minocycline was determined in 10 studies, including 1256 *Mycoplasma* and *Ureaplasma* isolates [*M. hominis* (92 isolates) and *U. urealyticum* (988 isolates)]; the proportions were 18% (95% CI 3–66%) and 9% (95% CI 4–21%) in *M. hominis* and *U. urealyticum*, respectively, with substantial heterogeneity (I^2^ > 88%; *P* = 0.5) was observed between included studies (Table 1, Fig. 2). Also, significant publication bias was detected (Egger rank correlation test, *P* = 0.6886). To analyze the trends for changes in the rate of minocycline resistance in recent years, we performed a meta-regression analysis for changes in the proportion of minocycline resistance to *Mycoplasma/Ureaplasma* urogenital isolates over time (Fig. 3). According to the meta-regression, the minocycline resistance rate decreased over time (r = −0.015; 95% CI −0.066 to 0.035, *P* = 0.550). We performed a meta-regression analysis for changes in the proportion of minocycline in male/female ratio to *Mycoplasma*/*Ureaplasma* urogenital isolates. According to the meta-regression, the minocycline resistance rate increased in male/female ratio (r = −0.009; 95% CI −0.030 to 0.012, *P* = 0.396). Among 7 countries reporting resistance data for tetracycline, 5 (55.5%) countries (Cuba, Iraq, UK, and USA) reported that ≥ 10% of isolates had minocycline resistance. There was a statistically significant difference in the minocycline resistance rates between different countries (*P* < 0.01) (Table 1). There was a statistically significant difference in the minocycline resistance rates between different continents (*P* < 0.02), and this rate was higher in North America than South America (33% vs 15%), Europe (33% vs 12%), and Asia (33% vs 10%). No significant difference was found in the AST method (*P* = 0.63) (Table 1).

## Discussion

*Mycoplasma* and *Ureaplasma* have been identified as important human pathogens of considerable urogenital problems such as non-gonococcal urethritis (NUG), cervicitis, and PID [45]. This family of bacteria (*Mycoplasmataceae*) remains understudied compared with other etiological agents of urinary tract infection (UTI), with less global financial support. In another hand, the innate absence of cell wall in *Mycoplasma* and *Ureaplasma* have led to intrinsic resistance to beta-lactams (one of the most routinely used antibiotics which act on cell wall) and limits the options for chemical therapy [46]. Also, innate resistance to lincosamides has been seen in *Ureaplasma* spp. Furthermore, *Mycoplasma* and *Ureaplasma* have resistance to sulphonamides, trimethoprim, and rifampicin [46].

Alternatively, tetracyclines, fluoroquinolones, and macrolides are widely used as effective anti-*Mycoplasma* agents [47]. Tetracyclines are ‘broad-spectrum’ antibiotics and have potent bacteriostatic activity against different pathogens, even *Mycoplasma* and *Ureaplasma* [48, 49]. While tetracyclines are divided into three generations, they have similar action mechanisms: first-generation, such as conventional tetracycline, which gets from biosynthesis. Second-generation like doxycycline and minocycline are semi-synthetic with improved properties and a wider spectrum of activity [50]. Finally, tigecycline is synthetic and located in third-generation with the highest and widest activity against gram-positive and negative bacteria (mini). Regarding action mechanisms, they can be passing through the bacterial membrane for reaching to target. This category of antibiotics is one of the most commonest used drugs against different forms of mycoplasmosis including urogenital or respiratory infections in adults, or animal infections on the farm as well [51]. The tetracycline antibiotics are capable of reversible binding to the 30S subunit of the bacterial ribosome, this interaction leads to the prevention of the association of aminoacyl-tRNA with the acceptor site in the bacterial ribosome and thus inhibition of the protein synthesis [51].

In this systematic review and meta-analysis, we calculated the resistance rate of tetracycline, doxycycline, and minocycline in *Mycoplasma* and *Ureaplasma* spp, recovered from human urogenital. The tetracyclines resistance was greatly variable in different literatures performed in various locations. Probably, the various consumption rates of these antibiotics in different parts of the world are the major reason for zonal differences in resistance rates of urogenital *Mycoplasma* and *Ureaplasma* species to the tetracyclines category [46]. Thus, reliable antibiotic susceptibility testing to obtain successful therapy is required. In another hand, *Mycoplasmas* and *Ureaplasma* like any other Mollicutes, are fastidious, thus their culture and routine antimicrobial susceptibility testing depend on specialized growth medium requirements. Therefore, commercial kits have been applied for detection and antimicrobial susceptibility testing [52, 53]. Undoubtedly, each commercial kit has special efficiency in antimicrobial susceptibility testing. On the other hand, a large number of available kits have not confirmed breakpoints by Clinical and Laboratory Standards Institute (CLSI)-recommended standard criteria for genital mycoplasmosis, and require confirming by other methods [54]. As a result, almost all urogenital mycoplasmosis infections are treated empirically [55]. Maybe, resistance heterogeneity and empirical therapy are two reasons for resistance development.

Our results demonstrated relatively high tetracyclines (including tetracycline, doxycycline, and minocycline) resistance rates in *M. hominis*, *U.* *urealyticum*, and *U. parvum*. It was also, the lowest resistance rate in *M. hominis*, *U*. *urealyticum*, and *U. parvum* had been seen to minocycline. Moreover, the highest rate of resistance among these three antibiotics is associated with tetracycline, most likely due to massive and frequent misuse of this antibiotic [56]. These results are in concordance with the previous meta-analysis [5]. While chemical therapy is capable to abolish urogenital mycoplasmosis, failed treatment makes persistent infections and resistant-strains expansion. Other studies showed that the tetracyclines-resistant rate is increasing and changing over time [46, 57]. Therefore, successful treatment with these antibiotics will be limited, more challenging, and costlier in further infections [45, 57].

Tetracyclines resistant bacteria applied genes encoding resistance which were placed on plasmids and transposons elements. These elements are mobile and make bacteria competent to horizontal gene transfer and resistance expansion among pathogens including *M. hominis*, *U.* *urealyticum*, and *U. parvum* [58, 59]. The development of tetracycline resistance in Mycoplasmas is associated with two major mechanisms including an active drug efflux pump and the production of ribosome-protecting proteins by the *tet* (M) gene [60]. This gene is located on transposon and encodes tetM protein (homolog of bacterial elongation factors) which causes conformational changes in the 30S ribosomal subunit and inhibition of tetracycline binding [47]. Moreover, several other mechanisms contribute to tetracyclines resistance including a decreased influx of antibiotics into the cell, antibiotic modification by enzymes, and target site modification by a mutation in the tetracycline-binding unit of 16S rRNA [5, 51, 56].

Recently, tetracyclines resistance Mycoplasmas have increased greatly; this phenomenon is a growing problem worldwide. Undoubtedly, an uninterrupted effort is required to urgently discover new therapeutic options, which lead to improved treatment. Raising knowledge in resistance mechanisms to tetracyclines and the development of chemical substances provided a potent capacity to introduce new effective generations of tetracyclines. However, not only the new generation of tetracyclines but also the international monitoring system exposing reliable antimicrobial susceptibility testing is crucial.

Interpretation of the present study results is constrained by several limitations. Firstly, variety in sample size can affect the analysis. But, the problem was solved by calculating and reporting of relative weight for each study. Another limitation is excluding the published articles other than the English language, therefore some articles would have been missed.

## Conclusions

In summary, regarding the present meta-analysis, the overall resistance rate to tetracyclines (tetracyclines, doxycycline, and minocycline) is relatively high in urogenital Mycoplasmas including *M. hominis*, *U. urealyticum*, and *U. parvum.* Also, the geographical variations in the prevalence of resistance showed that resistance in America continent is higher in comparison to other continents. These geographical variations demonstrate the importance of regional antibiotic susceptibility testing to control and eliminate infections caused by resistant strains.

### Supplementary Information

**Additional file 1. **Basic information of the included studies.

## Data Availability

All the data in this review are included in the manuscript. The data of the study is with the corresponding author of the article and can be made available on request.

## Abbreviations

PID: Pelvic inflammatory disease
PRISMA: Preferred reporting items for systematic reviews and meta analyses
AST: Antimicrobial susceptibility testing
CI: Confidence interval
NUG: Non-gonococcal urethritis
UTI: Urinary tract infection
CLSI: Clinical and Laboratory Standards Institute

## References

[1] Kong Y, Qiao Y, Song J, Ruan Z, Fei C, Huang J (2016). Comparative analysis of male and female populations on prevalence and antibiotic resistance of *Mycoplasma*
*hominis* in China, 2005–2014. J Glob Antimicrob Resist.

[2] Zhang W, Li L, Zhang X, Fang H, Chen H, Rong C (2021). Infection prevalence and antibiotic resistance levels in *Ureaplasma*
*urealyticum* and *Mycoplasma*
*hominis* in gynecological outpatients of a tertiary hospital in China from 2015 to 2018. Can J Infect Dis Med Microbiol.

[3] Vargović M, Pasini M, Papić N, Andrašević S, Markotić A, Butić I (2014). Antimicrobial susceptibility of *Ureaplasma*
*urealyticum* and *Mycoplasma*
*hominis*. Sex Transm Infect.

[4] Riedel S, Morse SA, Mietzner TA, Miller S (2019). Jawetz Melnick & Adelbergs medical microbiology 28 E.

[5] Ahmadi MH (2021). Resistance to tetracyclines among clinical isolates of *Mycoplasma*
*hominis* and Ureaplasma species: a systematic review and meta-analysis. J Antimicrob Chemother.

[6] Skiljevic D, Mirkov D, Vukicevic J (2016). Prevalence and antibiotic susceptibility of *Mycoplasma*
*hominis* and *Ureaplasma*
*urealyticum* in genital samples collected over 6 years at a Serbian university hospital. Indian J Dermatol Venereol Leprol.

[7] Tadongfack TD, Nitcheu ILS, Keubo FRN, Mutarambirwa HD, Tedjieu RH, Tatang CT (2020). Epidemiology, prevalence and antimicrobial susceptibility of sexually transmitted *Mycoplasma*
*hominis* and *Ureaplasma*
*urealyticum* infections in Dschang, West Cameroon. Microbiol Res J Int.

[8] Waites KB, Schelonka RL, Xiao L, Grigsby PL, Novy MJ (2009). Congenital and opportunistic infections: Ureaplasma species and *Mycoplasma**hominis*. Seminars in fetal and neonatal medicine.

[9] De Francesco MA, Caracciolo S, Bonfanti C, Manca N (2013). Incidence and antibiotic susceptibility of *Mycoplasma*
*hominis* and *Ureaplasma*
*urealyticum* isolated in Brescia, Italy, over 7 years. J Infect Chemother.

[10] Lee JY, Yang JS (2020). Prevalence and antimicrobial susceptibility of *Mycoplasma*
*hominis* and Ureaplasma species in nonpregnant female patients in South Korea indicate an increasing trend of pristinamycin-resistant isolates. Antimicrob Agents Chemother.

[11] Xie X, Zhang J (2006). Trends in the rates of resistance of *Ureaplasma*
*urealyticum* to antibiotics and identification of the mutation site in the quinolone resistance-determining region in Chinese patients. FEMS Microbiol Lett.

[12] Furneri PM, Rappazzo G, Musumarra MP, Tempera G, Roccasalva LS (2000). Genetic basis of natural resistance to erythromycin in Mycoplasma hominis. J Antimicrob Chemother.

[13] Kechagia N, Bersimis S, Chatzipanagiotou S (2008). Incidence and antimicrobial susceptibilities of genital mycoplasmas in outpatient women with clinical vaginitis in Athens, Greece. J Antimicrob Chemother.

[14] Waites KB, Crabb DM, Bing X, Duffy LB (2003). In vitro susceptibilities to and bactericidal activities of garenoxacin (BMS-284756) and other antimicrobial agents against human mycoplasmas and ureaplasmas. Antimicrob Agents Chemother.

[15] Moher D, Liberati A, Tetzlaff J, Altman DG, med PGJP (2009). Preferred reporting items for systematic reviews and meta-analyses: the PRISMA statement. Plos Med.

[16] Modesti PA, Reboldi G, Cappuccio FP, Agyemang C, Remuzzi G, Rapi S (2016). Panethnic differences in blood pressure in Europe: a systematic review and meta-analysis. PLoS ONE.

[17] Schwarzer GJRn. meta: an R package for meta-analysis. 2007; 7(3): 40–5.

[18] Team RCJhwR-po. R: a language and environment for statistical computing. R Foundation for Statistical Computing, Vienna, Austria. 2013.

[19] Evans RT, Taylor-Robinson D (1978). The incidence of tetracycline-resistant strains of *Ureaplasma*
*urealyticum*. J Antimicrob Chemother.

[20] Magalhaes M, Veras A (1984). Minocycline resistance among clinical isolates of *Ureaplasma*
*urealyticum*. J Infect Dis.

[21] Roberts MC, Kenny GE (1986). TetM tetracycline-resistant determinants in *Ureaplasma*
*urealyticum*. Pediatr Infect Dis.

[22] Busolo F, Conventi L (1988). In vitro activity of antibiotics against *Ureaplasma*
*urealyticum* and Chlamydia trachomatis strains from patients with nongonococcal urethritis. Eur J Clin Microbiol Infect Dis.

[23] Cakan H, Polat E, Kocazeybek B, Ocal P, Cepni I, Aslan M (2003). Assessment of antibiotic susceptibility of *Ureaplasma*
*urealyticum* from prostitutes and outpatient clinic patients using the E-test and agar dilution method. Chemotherapy.

[24] Huang C, Liu Z, Lin N, Tu Y, Li J, Zhang D (2003). Susceptibility of mixed infection of *Ureaplasma*
*urealyticum* and *Mycoplasma*
*hominis* to seven antimicrobial agents and comparison with that of *Ureaplasma*
*urealyticum* infection. J Huazhong Univ Sci Technol Med Sci.

[25] Xie X, Zhang J (2006). Trends in the rates of resistance of Ureaplasma urealyticum to antibiotics and identification of the mutation site in the quinolone resistance-determining region in Chinese patients. FEMS Microbiol Lett.

[26] Chang-tai Z, Zhong-yi H, Chun-lei D, Chang-song Z, Mei-zhen W, Yang L (2011). Investigation of *Ureaplasma*
*urealyticum* biovars and their relationship with antimicrobial resistance. Indian J Med Microbiol.

[27] Govender S, Gqunta K, le Roux M, de Villiers B, Chalkley LJ (2012). Antibiotic susceptibilities and resistance genes of Ureaplasma parvum isolated in South Africa. J Antimicrob Chemother.

[28] Díaz L, Cabrera LE, Fernández T, Ibáñez I, Torres Y, Obregón Y (2013). Frequency and antimicrobial sensitivity of *Ureaplasma*
*urealyticum* and *Mycoplasma*
*hominis* in patients with vaginal discharge. MEDICC Rev.

[29] Pignanelli S, Pulcrano G, Iula VD, Zaccherini P, Testa A, Catania MR (2014). In vitro antimicrobial profile of *Ureaplasma*
*urealyticum* from genital tract of childbearing-aged women in Northern and Southern Italy. APMIS.

[30] Ye G, Jiang Z, Wang M, Huang J, Jin G, Lu S (2014). The resistance analysis of *Ureaplasma*
*urealyticum* and *Mycoplasma*
*hominis* in female reproductive tract specimens. Cell Biochem Biophys.

[31] Tzimoula K, Maria E, Eudoxia D, Georgia G, Angeliki M, Nikolaos M (2015). Detection of the tetM resistance determinant among phenotypically sensitive Ureaplasma species by a novel real-time PCR method. Diagn Microbiol Infect Dis.

[32] Fernández J, Karau MJ, Cunningham SA, Greenwood-Quaintance KE, Patel R (2016). Antimicrobial susceptibility and clonality of clinical ureaplasma isolates in the United States. Antimicrob Agents Chemother.

[33] He M, Xie Y, Zhang R, Gao S, Xu G, Zhang L (2016). Prevalence and antimicrobial resistance of Mycoplasmas and Chlamydiae in patients with genital tract infections in Shanghai, China. J Infect Chemother.

[34] Al-Dahmoshi HOM, Al-Sharef HK, Kadhum SA, Al-Asskar JA, Al-Khafaji NS, Karkosh AS (2017). Rapid detection and antibiotic susceptibility of genital mycoplasma isolated from male with urethritis and prostatitis, Iraq. J Pure Appl Microbiol.

[35] Al-khafaji GK (2017). Susceptibility and antimicrobial resistance of genital *Ureaplasma*
*Parvum*. Nano Biomed Eng.

[36] Valentine-King MA, Brown MB (2017). Antibacterial resistance in ureaplasma species and *Mycoplasma*
*hominis* isolates from urine cultures in college-aged females. Antimicrob Agents Chemother.

[37] Meygret A, Le Roy C, Renaudin H, Bébéar C, Pereyre S (2018). Tetracycline and fluoroquinolone resistance in clinical Ureaplasma spp. and *Mycoplasma*
*hominis* isolates in France between 2010 and 2015. J Antimicrob Chemother..

[38] Al-Dahmoshi HO, Alwash MS, Chabuck SI, Al-Khafaji NS, Jabuk SI (2019). Antimicrobial susceptibility patterns of genital mycoplasma infections in pregnancy and spontaneous abortion. Res J Pharm Technol.

[39] Song T, Huang J, Liu Z, Zhang Y, Kong Y, Ruan Z (2019). Antibiotic susceptibilities and genetic variations in macrolide resistance genes of Ureaplasma spp. isolated in China. New Microbiol.

[40] Ahouga Voufo R, Maïdadi MF, Mbah EC, Esemu LF, Fouodji HP, Molu JP (2020). STUDY on the gender prevalence and sensitivity of urogenital mycoplasmas to antibiotics in Yaounde, Cameroon. Sci Afr.

[41] Baraïka MA, Onanga R, Bivigou-Mboumba B, Mabika-Mabika A, Bisvigou UJ, Touré Ndouo FS (2020). Prevalence and antimicrobial susceptibility profile of *Mycoplasma*
*hominis* and *Ureaplasma*
*urealyticum* in female population, Gabon. J Appl Biol Biotechnol..

[42] Boujemaa S, Mlik B, Mardassi H, Mardassi BBA (2020). Clonal spread of tetracycline resistance among *Mycoplasma*
*hominis* clinical strains, Tunisia. Infect Drug Resist.

[43] Doroftei B, Ilie OD, Armeanu T, Anton E, Scripcariu I, Maftei R (2021). The prevalence of *Ureaplasma*
*urealyticum* and *Mycoplasma*
*hominis* infections in infertile patients in the Northeast Region of Romania. Medicina (Kaunas, Lithuania).

[44] Rehman SU, Day J, Afshar B, Rowlands RS, Billam H, Joseph A (2021). Molecular exploration for *Mycoplasma*
*amphoriforme*, *Mycoplasma*
*fermentans* and Ureaplasma spp. in patient samples previously investigated for Mycoplasma pneumoniae infection. Access Microbiol.

[45] Daubenspeck JM, Totten AH, Needham J, Feng M, Balish MF, Atkinson TP (2020). Mycoplasma genitalium biofilms contain poly-GlcNAc and contribute to antibiotic resistance. Front Microbiol.

[46] Kokkayil P, Dhawan B (2015). Ureaplasma: current perspectives. Indian J Med Microbiol.

[47] Waites KB, Lysnyansky I, Bébéar CM. Emerging antimicrobial resistance in mycoplasmas of humans and animals. Mollicutes: molecular biology and pathogenesis. 2014:289–322.

[48] Boujemaa S, Mlik B, Ben Allaya A, Mardassi H, Mardassi BB (2020). Spread of multidrug resistance among *Ureaplasma*
*serovars*, Tunisia. Antimicrob Resist Infect Control.

[49] Cazanave C, Manhart L, Bébéar C (2012). Mycoplasma genitalium, an emerging sexually transmitted pathogen. Med Mal Infect.

[50] Markley JL, Fang L, Gasparrini AJ, Symister CT, Kumar H, Tolia NH (2019). Semisynthetic analogues of anhydrotetracycline as inhibitors of tetracycline destructase enzymes. ACS Infect Dis.

[51] Chernova O, Medvedeva E, Mouzykantov A, Baranova N, Chernov V (2016). Mycoplasmas and their antibiotic resistance: the problems and prospects in controlling infections. Acta Naturae.

[52] Morris DJ, Jones LC, Davies RL, Sands K, Portal E, Spiller OB (2020). MYCO WELL D-ONE detection of Ureaplasma spp. and *Mycoplasma*
*hominis* in sexual health patients in Wales. Eur J Clin Microbiol Infect Dis..

[53] Mao X-R, Wang R-C, Li R-J, Zhou C-R, Chen X-K, Cheng C-C (2020). An observational study: is N-acetylcysteine helpful in performance improvement of Mycoplasma IST2 testing through sample homogenization?. Can J Infect Dis Med Microbiol.

[54] Noh J-W, Kim K-B, Lee JH, Lee Y, Lee B-H, Kwon YD (2017). Breakpoints of the *Mycoplasma*
*hominis* and *Ureaplasma*
*urealyticum*. Yonsei Med J.

[55] Beeton ML, Spiller OB (2017). Antibiotic resistance among Ureaplasma spp. isolates: cause for concern?. J Antimicrob Chemother.

[56] Berçot B, Charreau I, Rousseau C, Delaugerre C, Chidiac C, Pialoux G (2021). High prevalence and high rate of antibiotic resistance of *Mycoplasma*
*genitalium* infections in men who have sex with men: a substudy of the ANRS IPERGAY pre-exposure prophylaxis trial. Clin Infect Dis.

[57] Dumke R, Spornraft-Ragaller P (2021). Antibiotic resistance and genotypes of *Mycoplasma*
*genitalium* during a resistance-guided treatment regime in a German university hospital. Antibiotics.

[58] Dai M, Lu J, Wang Y, Liu Z, Yuan Z (2012). In vitro development and transfer of resistance to chlortetracycline in *Bacillus*
*subtilis*. J Microbiol.

[59] Jahan M, Zhanel GG, Sparling R, Holley RA (2015). Horizontal transfer of antibiotic resistance from *Enterococcus*
*faecium* of fermented meat origin to clinical isolates of E. faecium and *Enterococcus*
*faecalis*. Int J Food Microbiol.

[60] Mardassi BBA, Aissani N, Moalla I, Dhahri D, Dridi A, Mlik B (2012). Evidence for the predominance of a single tet (M) gene sequence type in tetracycline-resistant *Ureaplasma*
*parvum* and *Mycoplasma*
*hominis* isolates from Tunisian patients. J Med Microbiol.

