# Supplementary material for: Tetracyclines resistance in Mycoplasma and Ureaplasma urogenital isolates derived from human: a systematic review and meta-analysis
Source: Ann Clin Microbiol Antimicrob. 2023 Sep 11;22:83. doi: 10.1186/s12941-023-00628-5 (PMC10496389; doi:10.1186/s12941-023-00628-5)
Supplement: Supplementary file 1 — Additional file 1. Basic information of the included studies. [file 12941_2023_628_MOESM1_ESM.docx]

| **First Author** | **Published Year** | **Country** | **Species of Bacterial Isolates** | **Total Number of Isolates** | **AST Group** | **Tetracycline** | **Doxycycline** | **Minocycline** |
| --- | --- | --- | --- | --- | --- | --- | --- | --- |
| Roberts[1] | 1986 | USA | *Ureaplasma urealyticum* | 63 | broth dilution method | 45 |  |  |
| Xie[2] | 2006 | China | *Ureaplasma urealyticum* | 804 | Mycoplasma IST2 (BioMerieux, Marcy L’etoile, France) | 83 | 57 |  |
| Ye[3] | 2013 | China | *Ureaplasma urealyticum* | 15594 | Mycoplasma assay kit from Biomerieux (France) | 557 | 379 |  |
| Ye[3] | 2013 | China | *Mycoplasma hominis* | 467 | Mycoplasma assay kit from Biomerieux (France) | 10 | 0 |  |
| Pignanelli[4] | 2013 | Italy | *Ureaplasma urealyticum* | 899 | Mycoplasma IST 2 kit (bioM erieux,Marcy-l’ Etoile, France | 26 | 15 |  |
| Song[5] | 2014 | China | *Mycoplasma hominis* | 42 | Mycoplasma IST 2 kit (bioMérieux, Marcy L’etoile, France), | 2 | 0 |  |
| Tzimoula[6] | 2014 | Greece | *Ureaplasma parvum* | 87 | (Mycoplasma IST 2; Biomerieux SA, Marcy l'Etoile, France). | 0 | 0 |  |
| Tzimoula[6] | 2014 | Greece | *Ureaplasma urealyticum,* | 12 | (Mycoplasma IST 2; Biomerieux SA, Marcy l'Etoile, France). | 0 | 0 |  |
| Valentine-King[7] | 2017 | USA | *Ureaplasma urealyticum* | 13 | microbroth dilution methods and agar dilution | 0 |  |  |
| Valentine-King[7] | 2017 | USA | *Ureaplasma parvum* | 60 | microbroth dilution methods and agar dilution | 1 |  |  |
| Valentine-King[7] | 2017 | USA | *Mycoplasma hominis* | 10 | microbroth dilution methods and agar dilution | 0 |  |  |
| Ahouga Voufo[8] | 2020 | Cameron | *Ureaplasma urealyticum* | 14 | MYCOFAST Evolution Kit 3 (EliTech Microbio) |  | 8 |  |
| Ahouga Voufo[8] | 2020 | Cameron | *Mycoplasma hominis* | 4 | MYCOFAST Evolution Kit 3 (EliTech Microbio) |  | 0 |  |
| Al-Dahmoshi[9] | 2019 | Iraq | *Mycoplasma hominis* | 10 | Mycoplasma IES Plus Kit | 9 |  | 10 |
| Al-Dahmoshi[9] | 2019 | Iraq | *Ureaplasma urealyticum* | 23 | Mycoplasma IES Plus Kit | 20 |  | 22 |
| Al-Dahmoshi[10] | 2017 | Iraq | *Ureaplasma urealyticum* | 13 | Mycoplasma IES Plus Kit | 2 |  | 0 |
| Al-Dahmoshi[10] | 2017 | Iraq | *Mycoplasma hominis* | 13 | Mycoplasma IES Plus Kit | 0 |  | 0 |
| Al-khafaji[11] | 2017 | Iraq | *Ureaplasma parvum* | 35 | MIC(broth microdilution) | 12 | 3 |  |
| Baraïka[12] | 2020 | Senegal | *Ureaplasma urealyticum* | 180 | Mycoplasma IST2 Kit (bioMérieux) | 47 | 20 |  |
| Baraïka[12] | 2020 | Senegal | *Mycoplasma hominis* | 63 | Mycoplasma IST2 Kit (bioMérieux) | 28 | 8 |  |
| Boujemaa[13] | 2020 | Tunisia | *Mycoplasma hominis* | 65 | MIC(broth microdilution) | 22 | 0 |  |
| Busolo and Conventi[14] | 1998 | Italy | *Ureaplasma spp* | 35 | broth dilutions | 34 | 0 | 12 |
| Çakan[15] | 2003 | Turkey | *Ureaplasma urealyticum* | 30 | MIC(agar dilution) , E-test | 0 | 0 |  |
| Chalker[16] | 2021 | England | *Mycoplasma hominis* | 81 | MIC(broth microdilution) | 12 |  |  |
| Chang-tai[17] | 2011 | China | *Ureaplasma urealyticum* | 126 | MIC(broth microdilution) |  | 2 | 1 |
| Díaz[18] | 2013 | Cuba | *Ureaplasma urealyticum* | 154 | Mycoplasma System  Plus | 48 | 26 | 25 |
| Díaz[18] | 2013 | Cuba | *Mycoplasma hominis* | 50 | Mycoplasma System  Plus | 29 | 9 | 5 |
| Doroftei[19] | 2021 | Romania | *Ureaplasma urealyticum* | 50 | Mycoplasma IES | 4 |  | 1 |
| Evans[20] | 1978 | England | *Ureaplasma spp* | 141 | microdilution |  |  | 14 |
| Fernández[21] | 2016 | Spain | *Ureaplasma parvum* | 202 | MIC (broth microdilution) | 1 |  |  |
| Fernández[21] | 2016 | Spain | *Ureaplasma urealyticum* | 48 | MIC (broth microdilution) | 0 |  |  |
| Govender[22] | 2012 | South Africa | *Ureaplasma parvum* | 50 | MIC (broth microdilution) | 5 |  |  |
| He[23] | 2016 | china | *Ureaplasma urealyticum* | 294 | Mycoplasmas kit produced by Zhengzhou Biological En-gineering Co |  | 18 | 14 |
| He[23] | 2016 | China | *Mycoplasma hominis* | 19 | Mycoplasmas kit produced by Zhengzhou Biological En-gineering Co |  | 1 | 1 |
| He[23] | 2016 | China | *Ureaplasma urealyticum + Mycoplasma hominis* | 139 | Mycoplasmas kit produced by Zhengzhou Biological En-gineering Co |  | 17 | 17 |
| Huang[24] | 2003 | China | *Ureaplasma urealyticum* | 150 | kits were produced by Heima Bio-logical and Engineering Limited Company in Zhuhai~ Guangzhou Province |  | 8 | 9 |
| Huang[24] | 2003 | China | *Ureaplasma urealyticum + Mycoplasma hominis* | 42 | kits were produced by Heima Bio-logical and Engineering Limited Company in Zhuhai~ Guangzhou Province |  | 11 | 9 |
| Magalhaes[25] | 1984 | USA | *Ureaplasma urealyticum* | 52 | direct agar dilution |  |  | 17 |
| Meygret[26] | 2018 | France | *Mycoplasma hominis* | 183 | commercial kits, the S.I.R. Mycoplasma (Bio-Rad, Hercules, CA, USA - MYCOFAST RevolutioN kit (ELITechGroup, Puteaux, France | 27 |  |  |

1. Roberts, M.C. and G.E. Kenny, *TetM tetracycline-resistant determinants in Ureaplasma urealyticum.* Pediatr Infect Dis, 1986. **5**(6 Suppl): p. S338-40.

2. Xie, X. and J. Zhang, *Trends in the rates of resistance of Ureaplasma urealyticum to antibiotics and identification of the mutation site in the quinolone resistance-determining region in Chinese patients.* FEMS Microbiol Lett, 2006. **259**(2): p. 181-6.

3. Ye, G., et al., *The resistance analysis of Ureaplasma urealyticum and Mycoplasma hominis in female reproductive tract specimens.* Cell Biochem Biophys, 2014. **68**(1): p. 207-10.

4. Pignanelli, S., et al., *In vitro antimicrobial profile of Ureaplasma urealyticum from genital tract of childbearing-aged women in Northern and Southern Italy.* Apmis, 2014. **122**(6): p. 552-5.

5. Song, T., et al., *Antibiotic susceptibilities and genetic variations in macrolide resistance genes of Ureaplasma spp. isolated in China.* New Microbiol, 2019. **42**(4): p. 225-227.

6. Tzimoula, K., et al., *Detection of the tetM resistance determinant among phenotypically sensitive Ureaplasma species by a novel real-time PCR method.* 2015.

7. Valentine-King, M.A. and M.B. Brown, *Antibacterial Resistance in Ureaplasma Species and Mycoplasma hominis Isolates from Urine Cultures in College-Aged Females.* Antimicrob Agents Chemother, 2017. **61**(10).

8. Ahouga Voufo, R., et al., *STUDY on the gender prevalence and sensitivity of urogenital mycoplasmas to antibiotics in YAOUNDE, CAMEROON.* 2020.

9. Al-Dahmoshi, H.O., et al., *Antimicrobial susceptibility patterns of genital mycoplasma infections in pregnancy and spontaneous abortion.* 2019.

10. Al-Dahmoshi, H.O.M., et al., *Rapid detection and antibiotic susceptibility of genital mycoplasma isolated from male with urethritis and prostatitis, Iraq.* 2017.

11. Al-khafaji, G.K., *Susceptibility and antimicrobial resistance of genital Ureaplasma Parvum.* 2017.

12. Baraïka, M.A., et al., *Prevalence and antimicrobial susceptibility profile of mycoplasma hominis and ureaplasma urealyticum in female population, Gabon.* 2020.

13. Boujemaa, S., et al., *Clonal spread of tetracycline resistance among mycoplasma hominis clinical strains, Tunisia.* 2020.

14. Busolo, F. and L. Conventi, *In vitro activity of antibiotics against Ureaplasma urealyticum and Chlamydia trachomatis strains from patients with nongonococcal urethritis.* Eur J Clin Microbiol Infect Dis, 1988. **7**(3): p. 407-10.

15. Cakan, H., et al., *Assessment of antibiotic susceptibility of Ureaplasma urealyticum from prostitutes and outpatient clinic patients using the E-test and agar dilution method.* Chemotherapy, 2003. **49**(1-2): p. 39-43.

16. Rehman, S.U., et al., *Molecular exploration for Mycoplasma amphoriforme, Mycoplasma fermentans and Ureaplasma spp. in patient samples previously investigated for Mycoplasma pneumoniae infection.* 2021.

17. Chang-tai, Z., et al., *Investigation of Ureaplasma urealyticum biovars and their relationship with antimicrobial resistance.* Indian J Med Microbiol, 2011. **29**(3): p. 288-92.

18. Díaz, L., et al., *Frequency and antimicrobial sensitivity of Ureaplasma urealyticum and Mycoplasma hominis in patients with vaginal discharge.* MEDICC Rev, 2013. **15**(4): p. 45-7.

19. Doroftei, B., et al., *The Prevalence of Ureaplasma Urealyticum and Mycoplasma Hominis Infections in Infertile Patients in the Northeast Region of Romania.* Medicina (Kaunas), 2021. **57**(3).

20. Evans, R.T. and D. Taylor-Robinson, *The incidence of tetracycline-resistant strains of Ureaplasma urealyticum.* J Antimicrob Chemother, 1978. **4**(1): p. 57-63.

21. Fernández, J., et al., *Antimicrobial Susceptibility and Clonality of Clinical Ureaplasma Isolates in the United States.* Antimicrob Agents Chemother, 2016. **60**(8): p. 4793-8.

22. Govender, S., et al., *Antibiotic susceptibilities and resistance genes of Ureaplasma parvum isolated in South Africa.* J Antimicrob Chemother, 2012. **67**(12): p. 2821-4.

23. He, M., et al., *Prevalence and antimicrobial resistance of Mycoplasmas and Chlamydiae in patients with genital tract infections in Shanghai, China.* J Infect Chemother, 2016. **22**(8): p. 548-52.

24. Huang, C., et al., *Susceptibility of mixed infection of Ureaplasma Urealyticum and Mycoplasma Hominis to seven antimicrobial agents and comparison with that of Ureaplasma Urealyticum infection.* J Huazhong Univ Sci Technolog Med Sci, 2003. **23**(2): p. 203-5.

25. Magalhaes, M. and A. Veras, *Minocycline resistance among clinical isolates of Ureaplasma urealyticum.* 1984.

26. Meygret, A., et al., *Tetracycline and fluoroquinolone resistance in clinical Ureaplasma spp. and Mycoplasma hominis isolates in France between 2010 and 2015.* J Antimicrob Chemother, 2018. **73**(10): p. 2696-2703.
